# Laminar Motion of the Incompressible Fluids in Self-Acting Thrust Bearings with Spiral Grooves

**DOI:** 10.1155/2014/478401

**Published:** 2014-01-02

**Authors:** Cornel Velescu, Nicolae Calin Popa

**Affiliations:** ^1^Hydraulic Machinery Department, Mechanical Faculty, “Politehnica” University of Timisoara, Boulevard Mihai Viteazul No. 1, 300222 Timisoara, Romania; ^2^Center for Advanced and Fundamental Technical Research (CAFTR), Romanian Academy-Timisoara Branch, Boulevard Mihai Viteazul No. 24, 300223 Timisoara, Romania

## Abstract

We analyze the laminar motion of incompressible fluids in self-acting thrust bearings with spiral grooves with inner or external pumping. The purpose of the study is to find some mathematical relations useful to approach the theoretical functionality of these bearings having magnetic controllable fluids as incompressible fluids, in the presence of a controllable magnetic field. This theoretical study approaches the permanent motion regime. To validate the theoretical results, we compare them to some experimental results presented in previous papers. The laminar motion of incompressible fluids in bearings is described by the fundamental equations of fluid dynamics. We developed and particularized these equations by taking into consideration the geometrical and functional characteristics of these hydrodynamic bearings. Through the integration of the differential equation, we determined the pressure and speed distributions in bearings with length in the “pumping” direction. These pressure and speed distributions offer important information, both quantitative (concerning the bearing performances) and qualitative (evidence of the viscous-inertial effects, the fluid compressibility, etc.), for the laminar and permanent motion regime.

## 1. Introduction

Incompressible fluid motion in “self-acting thrust bearings with spiral grooves” (SATBESPIG) is a complex motion in thin layers [1–5] bounded by two solid surfaces in relative rotation one to another. The incompressible fluid motion in thin layers has as mathematical consequence the simplification and particularization of the general equations of motion. We made this simplification of the general equations of motion by neglecting the terms smaller by more than one order of magnitude [1–7] than the other terms. Starting from this observation, the paper approaches the theoretical study of incompressible fluid motion in SATBESPIG with inner and exterior pumping, working in the laminar regime.

Some authors have developed comparative theoretical and experimental studies concerning different aspects of spiral groove bearings functioning in the laminar regime, with equations for their functioning using magnetic fluids, cavitation phenomenon, and the geometry of the bearings with spiral/axial multiples grooves.

In [8, 9], the author studied the static and dynamic performances of the bearings with spiral grooves and inner and exterior pumping, working with compressed air. A comparative study of the theoretical and experimental results was presented.

The diversification and optimization of the geometry of the bearings with spiral or axial multiples of grooves were approached in [10–12]. In [13], the author analyzed the cavitation phenomenon theoretically and experimentally in bearings with spiral grooves working in mineral oil. The unfavorable consequences of the cavitation were evaluated.

We consider the theoretical results as a first step in our intention to approach the functionality of the hydrodynamic bearings in general and of the thrust bearings with spiral grooves in particular in the stationary/nonstationary turbulent regime. The viscous compressible/incompressible fluid motion can be approached by taking into consideration the thermo-viscous-inertial phenomena ensemble that exists in bearings.

## 2. Constructive Geometric and Cinematic Elements of the SATBESPIG

**Figure 1:**
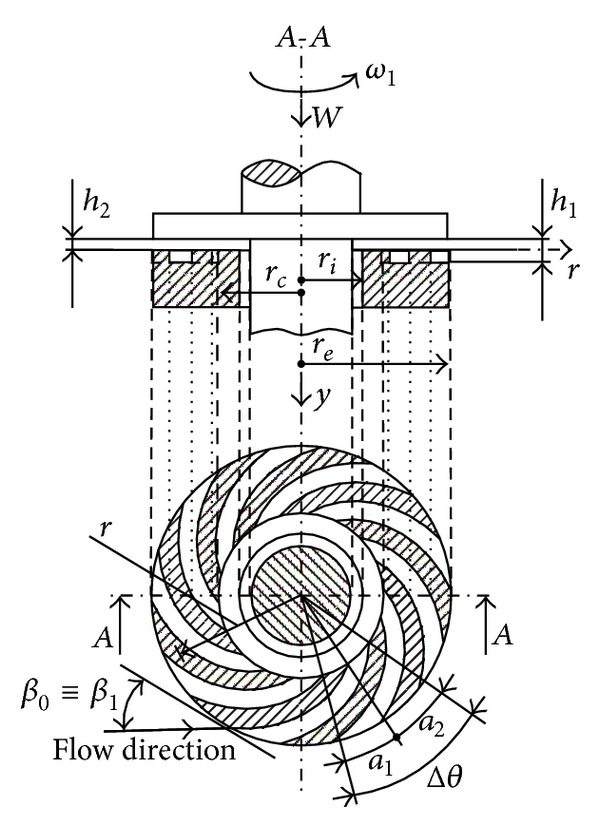
SATBESPIG and inner pumping.

**Figure 2:**
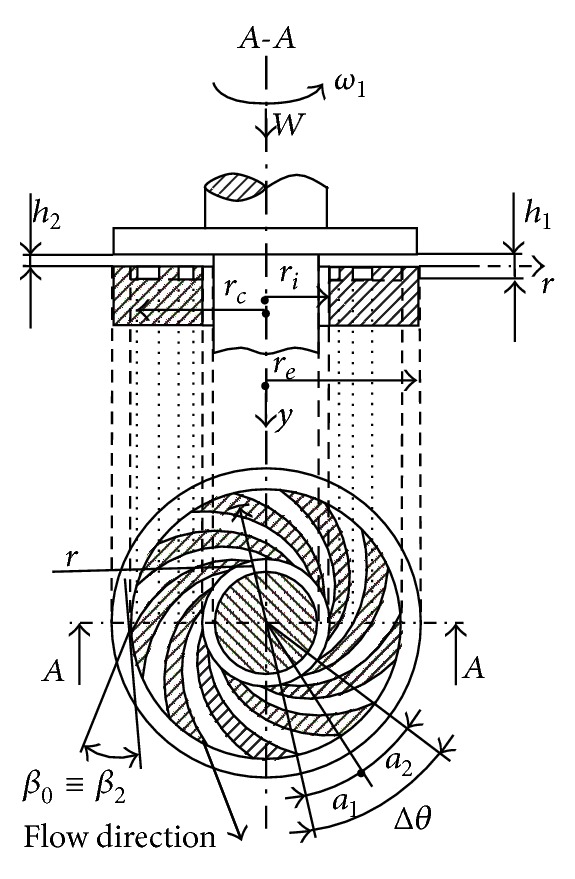
SATBESPIG and external pumping.

**Figure 3:**
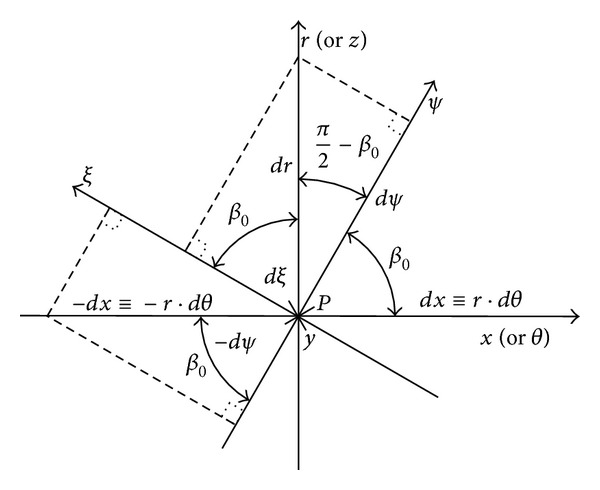
Relations between the general and local coordinate systems (in an arbitrary point, *P*). At point *P*, the coordinate *ξ* is normal to the *ψ* direction of the spiral channel.

**Figure 4:**
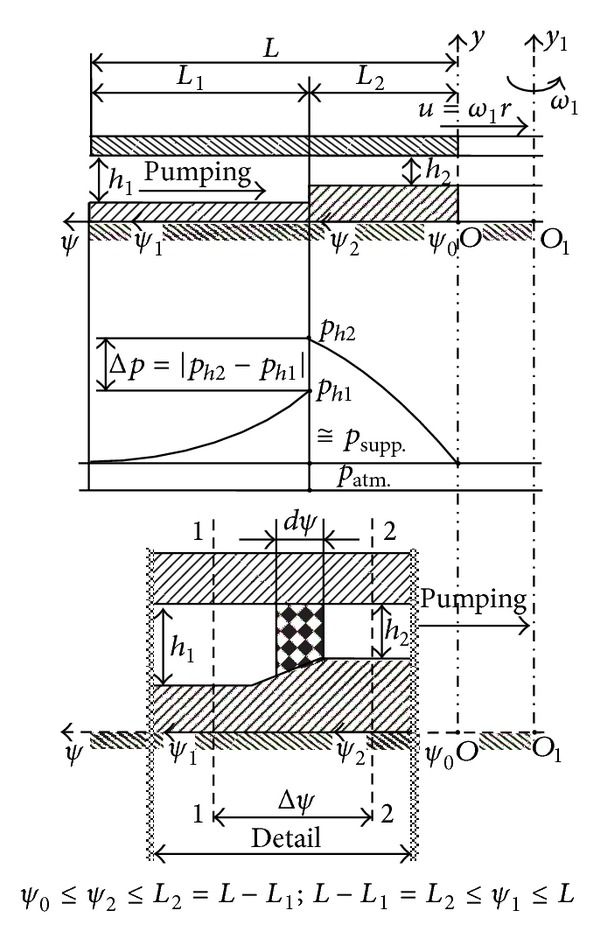
Radial step of the inner pumping bearing.

**Figure 5:**
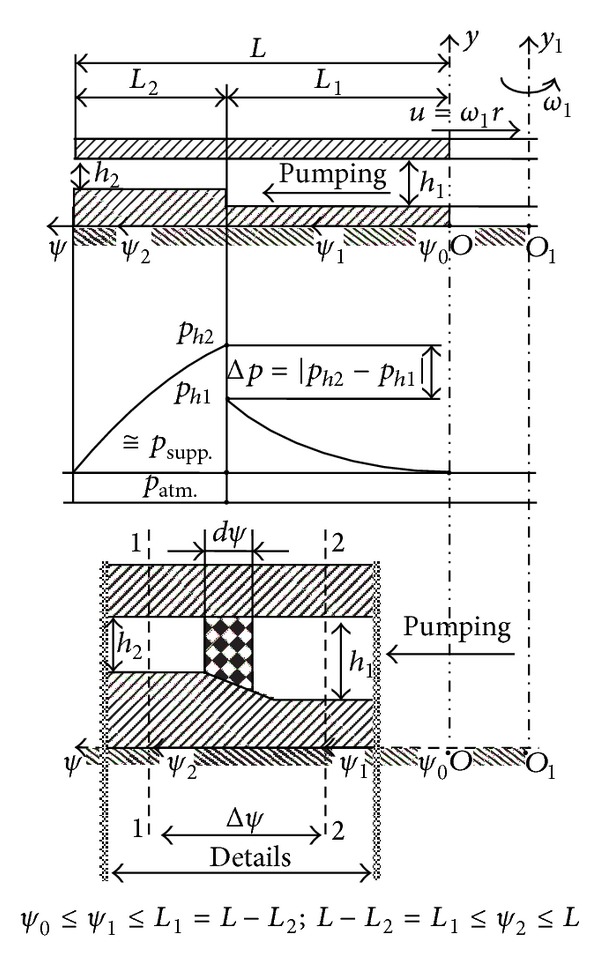
Radial step of the external pumping bearing.

The SATBESPIG (Figures 1 and 2) are special hydrodynamic bearings that have the specific effect of “autopumping” [1–3, 5, 7], which is why we are interested in the incompressible fluid motion in the laminar and permanent regime and, taking into consideration the influence of the inertial forces, in the length of the spiral groove (pumping direction *ψ* from Figures 3, 4, and 5). In the literature [4, 7, 10, 12–15], there are only a few studies and a small amount of theoretical research concerning this subject. These studies approach the incompressible fluid motion only in the radial direction (*r*) without taking into consideration the effects of the inertial forces.

In Figures 1 and 2, (i) *a*_1_ is the width of the spiral channel, measured on the circle arc contour; (ii) *a*_2_ is the width of the spiral threshold, measured on the circle arc contour; (iii) Δ*θ* is the center angle corresponding to a channel—threshold pair; (iv) *β*_0_ is the generator angle of the logarithmic spiral, which describes the form of the cannels; (v) *β*_1_ and *β*_2_ are the input and output angles, respectively, in and from the channel (according to the accepted notations from the general study of the hydraulic machineries); (vi) *ω*_1_ is the bearing angular rotation speed; (vii) *r*_*i*_ and *r*_*e*_ are the inner and external radius of the bearing, respectively; (viii) *r*_*c*_ is the radius marking the zone of the spiral channels; (ix) *r* is the current radius of the bearing; (x) *h*_1_ is the lubrication film height over the bearing channels; (xi) *h*_2_ is the lubrication film height over the bearing thresholds; (xii) *W* is the bearing load (the weight); and (xiii) by definition, *α* = *a*_1_/(*a*_1_ + *a*_2_).

With the geometrical dimensions in Figure 1 (or Figure 2) we can write Δ*θ* = 2*π*/*n*_*p*_, where *n*_*p*_ is the number of pairs channel—thresholds, *a*_1_ = *αr*Δ*θ*, *a*_2_ = (1 − *α*)*r*Δ*θ*, *a* = *a*_1_ + *a*_2_ = *r*Δ*θ*, and *ω*_2_ = 0 (the grooved surface is fixed).

## 3. Coordinate Systems, Control Volume, Speed Distributions, and Mass Flow Rate in Laminar Regime

To study the mathematical model, the coordinate systems and the control volume must be determined so as to define the speed distributions and the fluid mass flow rate [3, 5, 15]. The control volume (Vol) is the volume between the bearing surfaces, between the one channel surface and one consecutive threshold surface of the stator and the horizontal bottom surface of the rotor (Figures 1 and 2). In Figure 3, we show the general (*y*, *r*, *θ*) and local (*ψ*, *y*, *ξ*) coordinate systems used for the motion study.

Next, we start from the fact that the incompressible fluid laminar motion, between the two quasiparallel surfaces of the bearing, is described by the following speed profiles:

(1a) $$\begin{array}{c} u\left(y\right)≅-\frac{1}{2\eta }\frac{1}{r}\frac{\partial p}{\partial \theta }y\left(h-y\right)+\frac{y}{h}\omega_{1}r, \end{array}$$

(1b) $$\begin{array}{c} v_{n}\left(y\right)≅0, \end{array}$$

(1c) $$\begin{array}{c} w\left(y\right)≅-\frac{1}{2\eta }\frac{\partial p}{\partial r}y\left(h-y\right), \end{array}$$

(1d) $$\begin{array}{c} v_{\psi }\left(y\right)≅\frac{1}{2\eta r\sin\beta_{0}}\frac{\partial p}{\partial \psi }y\left(y-h\right)+\frac{y}{h}\omega_{1}r\cos\beta_{0}, \end{array}$$

(1e) $$\begin{array}{c} v_{\xi }\left(y\right)≅\frac{1}{2\eta r\sin\beta_{0}}\frac{\partial p}{\partial \xi }y\left(y-h\right)-\frac{y}{h}\omega_{1}r\sin\beta_{0}. \end{array}$$

In (1a)–(1e), (i) *u*(*y*) is the fluid speed component in the *x* (or *θ*) direction, (ii) *v*_*n*_(*y*) is the fluid speed component in the normal direction *y*, (iii) *w*(*y*) is the fluid speed component in the *z* (or *r*) direction, (iv) *v*_*ψ*_(*y*) is the fluid speed component in the *ψ* curb direction, (v) *v*_*ξ*_(*y*) is the fluid speed component in the *ξ* direction, (vi) *p* is the pressure in the fluid, (vii) *h* is the height of the lubrication film, and (viii) *η* is the fluid dynamical viscosity.

The speed distributions, given by (1a)–(1e), are typical for the noninertial motion case initially considered, meaning that these are parabolic speed profiles [3, 5, 6]. Observing the geometry of the bearings in Figures 1 and 2, it is possible to establish some functional mathematical relations between the coordinates. Thus, the following operational expressions can be found [3, 5]:

(2a) $$\begin{array}{c} \frac{\partial }{\partial r}=\cos\beta_{0}\frac{\partial }{\partial \xi }+\sin\beta_{0}\frac{\partial }{\partial \psi }, \end{array}$$

(2b) $$\begin{array}{c} \frac{\partial }{\partial x}\equiv \frac{1}{r}\frac{\partial }{\partial \theta }=-\sin\beta_{0}\frac{\partial }{\partial \xi }+\cos\beta_{0}\frac{\partial }{\partial \psi }, \end{array}$$

(2c) $$\begin{array}{c} \frac{\partial }{\partial \xi }=\cos\beta_{0}\frac{\partial }{\partial r}-\sin\beta_{0}\frac{1}{r}\frac{\partial }{\partial \theta }, \end{array}$$

(2d) $$\begin{array}{c} \frac{\partial }{\partial \psi }=\sin\beta_{0}\frac{\partial }{\partial r}+\cos\beta_{0}\frac{1}{r}\frac{\partial }{\partial \theta }. \end{array}$$

Using relation (1e), the fluid mass flow rate in the pumping direction *ψ* (Figures 4 and 5), further denoted by ${\dot{ℳ}}_{\psi }$, can be expressed by the integral representation [3, 5]:

(3) $$\begin{array}{c} {\dot{ℳ}}_{\psi }≅\overset{\theta +\Delta \theta }{\underset{\theta }{\int }}r_{0}\sin\beta_{0}d\theta \overset{h}{\underset{0}{\int }}\rho v_{\psi }\left(y\right)dy, \end{array}$$

where *ρ* is the fluid density and *r*_0_ is the “reference” radius (*r*_0_ ∈ [*r*_*i*_, …, *r*_*e*_], and further *r*_0_ will be denoted by *r*).

## 4. Differential Equation for Pressure Distribution in the *ψ* Direction 

Observing the bearing geometry (Figures 1 and 2), we admit that the angle Δ*θ* is infinitely small, meaning that there exist an infinite number of spiral channels. Given the relations (1e) and (3), the physical natural condition is that the mass flow rate ${\dot{ℳ}}_{\psi }$ is constant. With these conditions we obtain

(4) $$\begin{array}{c} {\dot{ℳ}}_{\psi }≅\Delta \theta \left[C\frac{\partial p}{\partial \psi }+Dr^{2}\right], \end{array}$$

where

(5a) $$\begin{array}{c} C=-\frac{\rho }{12\eta }\left[\alpha h_{1}^{3}+\left(1-\alpha \right)h_{2}^{3}\right], \end{array}$$

(5b) $$\begin{array}{c} D=\frac{\rho }{2}\omega_{1}\sin\beta_{0}\cos\beta_{0}\left[\alpha h_{1}+\left(1-\alpha \right)h_{2}\right]. \end{array}$$

The fluid mass conservation in the *ψ* direction can be expressed as follows:

(6) $$\begin{array}{c} \frac{\partial }{\partial \tau }\left(m\right)+\frac{\partial }{\partial \psi }\left({\dot{ℳ}}_{\psi }\right)\Delta \psi ≅0, \end{array}$$

where *τ* is the time, and the fluid mass *m*, contained in the control volume Vol, is given by the relation

(7) $$\begin{array}{c} m≅\Delta r\Delta \theta \rho r\left[\alpha h_{1}+\left(1-\alpha \right)h_{2}\right]. \end{array}$$

Or, using relations (4) and (7), (6) becomes

(8) ∂∂τ{[αh1+(1−α)h2]ρ} +sin2β01ψ∂∂ψ{C∂p∂ψ+D1sin2β0ψ2}≅0.

For the permanent motion regime, (8) becomes

(9) $$\begin{array}{c} \frac{1}{\psi }\frac{\partial }{\partial \psi }\left\{C\frac{\partial p}{\partial \psi }+\frac{1}{\sin^{2}\beta_{0}}D\psi^{2}\right\}≅0. \end{array}$$

Using nondimensional variables [1, 3–5, 7, 10, 14, 15], (8) can be written as

(10) $$\begin{array}{c} \frac{\partial }{\partial \zeta }\left[PH^{3}\left(K_{1}\zeta \frac{\partial P}{\partial \zeta }+K_{2}\Omega \Lambda \zeta^{2}H^{-2}\right)\right]-\sigma \zeta \frac{\partial }{\partial t}\left[PHK_{3}\right]≅0. \end{array}$$

For the stationary motion regime, (10) becomes [1, 2]

(11) $$\begin{array}{c} \frac{\partial }{\partial \zeta }\left[P\left(K_{1}\zeta \frac{\partial P}{\partial \zeta }+K_{2}\Omega \Lambda \zeta^{2}\right)\right]≅0. \end{array}$$

## 5. Integration of the Differential Equation of the Pressure Distribution in the *ψ* Direction

In the *ψ* direction, the fluid film *h* varies rapidly from *h*_1_ to *h*_2_, at the frontier *r*≅*r*_*c*_ (Figures 4 and 5). The existing radial step with length in the pumping direction *ψ* produces a pressure jump from *p*_*h*1_ to *p*_*h*2_. This pressure jump has different values as a function of (i) the flow regime through bearing (laminar, transition, or turbulent regime), (ii) the value of the rapport *h*_1_/*h*_2_, (iii) the fact that we take (or not) into consideration the inertial forces, and (iv) the fluid type [2, 3, 5, 16, 17].

Integrating differential equation (9) and observing the limit conditions for pressures [3, 5] and the notations from Figures 4 and 5 (*p*_supp._ is the supply pressure of the lubrication fluid and *p*_atm._ is the atmospheric pressure), we obtain the mathematical relations for the pressure distributions in the SATBESPIG:

(12) p(ψ) ≅ph2+ψ−(L2+ψ0)ψ0−(L2+ψ0)  ×{(psupp.−ph2)−2ηcosβ0ω1h22sinβ0[ψ03−(L2+ψ0)3]}  +2ηcosβ0ω1h22sinβ0·[ψ3−(L2+ψ0)3].

Relation (12) presents the pressure distribution in the laminar and permanent flow regime in the smooth region of the inner pumping bearing surface, where *h* = *h*_2_. Consider

(13) p(ψ) ≅psupp.+ψ−(L+ψ0)(L2+ψ0)−(L+ψ0)  ×{(ph1−psupp.)−2ηcosβ0ω1[αh1+(1−α)h2]sinβ0[αh13+(1−α)h23]   ×[(L2+ψ0)3−(L+ψ0)3]}  +2ηcosβ0ω1[αh1+(1−α)h2]sinβ0[αh13+(1−α)h23][ψ3−(L+ψ0)3].

Relation (13) presents the pressure distribution in the region with spiral channels of the inner pumping bearing surface, where *h* = *h*_1_.

In a similar way, for the SATBESPIG with exterior pumping (Figure 5), we obtain

(14) p(ψ)≅ph2+ψ−(L1+ψ0)(L+ψ0)−(L1+ψ0) ×{(psupp.−ph2)−2ηcosβ0ω1h22sinβ0[(L+ψ0)3−(L1+ψ0)3]} +2ηcosβ0ω1h22sinβ0[ψ3−(L1+ψ0)3].

Relation (14) presents the pressure distribution in the laminar and permanent flow regime in the smooth region of the external pumping bearing surface, where *h* = *h*_2_. Consider

(15) p(ψ) ≅psupp.+ψ−ψ0(L1+ψ0)−ψ0  ×{(ph1−psupp.)−2ηcosβ0ω1[αh1+(1−α)h2]sinβ0[αh13+(1−α)h23]    ×[(L1+ψ0)3−ψ03]}  +2ηcosβ0ω1[αh1+(1−α)h2]sinβ0[αh13+(1−α)h23][ψ3−ψ03].

Relation (15) presents the pressure distribution in the laminar and permanent motion regime in the spiral grooves region of the bearing surface with exterior pumping, where *h* = *h*_1_.

In the relations (12)–(15), all the constants are known (for a designed and realized SATBESPIG), the exception being the extreme pressures *p*_*h*1_ and *p*_*h*2_. If we do not take into consideration the influence of the inertial forces, then the pressures *p*_*h*1_ and *p*_*h*2_ are equal. Thus, *p*_*h*1_ ≡ *p*_*h*2_, Δ*p* = *p*_*h*2_ − *p*_*h*1_ = 0.

## 6. Calculus Relations for the Extreme Pressures *p*_*h*1_ and *p*_*h*2_

To express the pressure distribution in the *ψ* direction and the extreme pressures *p*_*h*1_ and *p*_*h*2_, we must analyze the liquid motion in the fluid film existing between the quasiparallel surfaces of the SATBESPIG [1–3, 5, 7]. On the other hand, the inertial effects (which considerably influence the extreme pressures *p*_*h*1_ and *p*_*h*2_) exist on all the surfaces of the SATBESPIG in the *ψ* direction, but the maximum effect is concentrated in the zone of the radial step, *r*≅*r*_*c*_ (Figures 4 and 5) [2, 3, 5, 16, 17].

Some theoretical results concerning the motion of liquids in similar bearings with the consideration of the influence of inertial forces have been presented in the literature [3, 5–7, 14]. It is possible to demonstrate [3, 5] that, for the case of the stationary motion regime and when only the smooth surface is in a rotation with *n*_1_ = constant [rot/min], the equation that describes the viscous fluid motion in *ψ* direction is

(16) ρψddψ(α0Qψ2hψ2Δθ2−γQψω1Δθcosβ0sinβ0+βω12ψ2cos2β0sin2β0h) +2ρδQψω1sinβ0ψ2Δθcosβ0−3ρβω12h+ρα0Qψ2hψ4Δθ2 −ργQψω1cosβ0ψ2Δθsinβ0+ρβω12cos2β0sin2β0h+1ψdpdψh +12ηh(QψhψΔθ−ω1ψcosβ02sinβ0)≅0,

where

(17a) Qψ=Uav.h≅−h312ηrsinβ0∂p∂ψ+h2ω1rcosβ0=const.  (volumic  flow  rate),

(17b) Uav.=1h∫0hvψ(y)·dy=average  speed  in  the  fluid  film  by  the  curb  direction  ψ,

(17c) $$\begin{array}{c} \alpha_{0}=\frac{6}{5},\;\;\beta =\frac{2}{15},\;\;\gamma =\frac{1}{5},\;\;\delta =\frac{1}{10}. \end{array}$$

The constructive angle of spiral groove is *β*_0_ = 17° [3, 5, 14].

Calculating the derivative of the relation (16) versus the variable *ψ* and taking into consideration the constants from above, the following expression can be found:

(18) dpdψ≅(ρα0Qψ2h3ψ2Δθ2−ρβω12ψ2hcos2β0sin2β0)dhdψ+ρα0Qψ2h2ψ3Δθ2 +3ρβω12ψ(1−cos2β0sin2β0)−2ρδQψω1sinβ0hψΔθcosβ0 +ργQψω1cosβ0hψΔθsinβ0−12ηQψh3Δθ+6ηω1ψ2cosβ0h2sinβ0.

We obtain a similar but more precise relation to (18) by introducing a supplementary coercive term [1, 2, 4, 9]. In this case, relation (18) becomes

(19) dpdψ≅[ρQψ2h3ψ2Δθ2(α0+εψ)]dhdψ−ρβω12ψ2hcos2β0sin2β0dhdψ +ρα0Qψ2h2ψ3Δθ2+3ρβω12ψ(1−cos2β0sin2β0) −2ρδQψω1sinβ0hψΔθcosβ0+ργQψω1cosβ0hψΔθsinβ0−12ηQψh3Δθ +6ηω1ψ2cosβ0h2sinβ0,

where *ε* = 2/15 [1, 2, 4].

Integrating the differential equation (19), for both regions of the SATBESPIG, where *h* = *h*_1_ = const. and *h* = *h*_2_ = const. (Figures 4 and 5), we obtain the calculus relations for the pressures *p*_*h*1_ and *p*_*h*2_:

(20) ph1≅psupp.+ρα0Qψ22h12Δθ2[1(L+ψ0)2−1(L2+ψ0)2] +32ρβω12(1−cos2β0sin2β0)[(L2+ψ0)2−(L+ψ0)2] −2ρδQψω1sinβ0h1Δθcosβ0lnL2+ψ0L+ψ0 +ργQψω1cosβ0h1Δθsinβ0lnL2+ψ0L+ψ0 +12ηQψh13Δθ[(L+ψ0)−(L2+ψ0)] −2ηω1cosβ0h12sinβ0·[(L+ψ0)3−(L2+ψ0)3],ph2≅psupp.−ρα0Qψ22h22Δθ2[1(L2+ψ0)2−1ψ02] +32ρβω12(1−cos2β0sin2β0)[(L2+ψ0)2−ψ02] +2ρδQψω1sinβ0h2Δθcosβ0lnψ0L2+ψ0 −ργQψω1cosβ0h2Δθsinβ0lnψ0L2+ψ0 +12ηQψh23Δθ[ψ0−(L2+ψ0)] +2ηω1cosβ0h22sinβ0[(L2+ψ0)3−ψ03].

These relations are valid for the SATBESPIG with inner pumping.

In a similar way, for the SATBESPIG with exterior pumping, we obtain

(21) ph1≅psupp.+ρα0Qψ22h12Δθ2[1ψ02−1(L1+ψ0)2] +32ρβω12(1−cos2β0sin2β0)[(L1+ψ0)2−ψ02] +2ρδQψω1sinβ0h1Δθcosβ0lnψ0L1+ψ0 −ργQψω1cosβ0h1Δθsinβ0lnψ0L1+ψ0 +12ηQψh13Δθ[ψ0−(L1+ψ0)] −2ηω1cosβ0h12sinβ0[ψ03−(L1+ψ0)3],

(22) ph2≅psupp.+ρα0Qψ22h22Δθ2[1(L+ψ0)2−1(L1+ψ0)2] +32ρβω12(1−cos2β0sin2β0)[(L1+ψ0)2−(L+ψ0)2] −2ρδQψω1sinβ0h2Δθcosβ0lnL1+ψ0L+ψ0 +ργQψω1cosβ0h2Δθsinβ0lnL1+ψ0L+ψ0 +12ηQψh23Δθ[(L+ψ0)−(L1+ψ0)] +2ηω1cosβ0h22sinβ0[(L1+ψ0)3−(L+ψ0)3].

In relations (20)–(22), all the variables are known except the volumetric flow rate *Q*_*ψ*_.

## 7. Calculus Relation for the Fluid Volumetric Flow Rate *Q*_*ψ*_

The calculus relation for the fluid volumetric flow rate *Q*_*ψ*_ will be established using differential equation (19) again. To find the calculus relation, at the position of the radial step of the bearing we suppose that *dh*/*dψ* ≠ 0. So, in this zone of the bearing the inertial effects are dominant in comparison to the viscous effects. In other words, in the radial step zone of the bearing, the liquid moves approximately like an ideal but not viscous fluid [3, 5].

Integrating (19) in the vicinity of the radial step of the bearing grooved surface (Figures 4 and 5), we obtain a relation between *p*_*h*1_, *p*_*h*2_, and *Q*_*ψ*_:

(23) Δp=ph1−ph2≅ρQψ2ψc2Δθ2(α0+εψc)(12h22−12h12) −ρβω12ψc2cos2β0sin2β0lnh1h2,

where *ψ*_*c*_ is the length measured by the *ψ* coordinate corresponding to the grooves radius of the profiled surface, *r*_*c*_ (Figures 4 and 5).

Relation (23) has some limits, especially at high values of *h*_1_/*h*_2_, when *h*_2_ → 0, or, in other words, at the heavy regimes for the bearing functionality. If we know the other variables, including the extreme pressures *p*_*h*1_ and *p*_*h*2_, relation (23) offers the flow rate *Q*_*ψ*_.

Therefore, using (20) and then (21), (22), and (23), we obtain a typical second degree algebraic equation, (24), from which we obtain the fluid flow rate *Q*_*ψ*_:

(24) ρQψ2Δθ2{α0[12h12(1(L+ψ0)2−1(L2+ψ0)2)     +12h22(1(L2+ψ0)2−1ψ02)]   −1ψc2(α0+εψc)(12h22−12h12)} +QψΔθ·[ργω1cosβ0sinβ0(1h1lnL2+ψ0L+ψ0+1h2lnψ0L2+ψ0)    −2ρδω1sinβ0cosβ0(1h1lnL2+ψ0L+ψ0+1h2lnψ0L2+ψ0)    +12η(L−L2h13+L2h23)]−T1+T2≅0,

where

(25) T1=32ρβω12(1−cos2β0sin2β0)[(L+ψ0)2−ψ02],T2=ρβω12ψc2cos2β0sin2β0lnh1h2−2ηω1cosβ0sinβ0 ×{1h12[(L+ψ0)3−(L2+ψ0)3]   +1h22[(L2+ψ0)3−ψ03]}.

Relation (24) is valid for the SATBESPIG with inner pumping.

For the SATBESPIG with exterior pumping, we obtain a similar relation:

(28) ρQψ2Δθ2{α0[12h22(1(L+ψ0)2−1(L1+ψ0)2)     −12h12(1ψ02−1(L1+ψ0)2)]   +12ψc2(α0+εψc)(1h22−1h12)} +QψΔθ·[ργω1cosβ0sinβ0(1h1lnψ0L1+ψ0+1h2lnL1+ψ0L+ψ0)    −2ρδω1sinβ0cosβ0(1h1lnψ0L1+ψ0+1h2lnL1+ψ0L+ψ0)    +12η(L−L1h23+L1h13)]−T1−T3≅0,

where

(29) T3=ρβω12ψc2cos2β0sin2β0lnh1h2 −2ηω1cosβ0sinβ0{1h12[ψ03−(L1+ψ0)3]  +1h22[(L1+ψ0)3−(L+ψ0)3]}.

Both (24) and (28) are classical algebraic equations of second degree in *Q*_*ψ*_. If we denote by *Q*_*ψ*_^*I*^ and *Q*_*ψ*_^*II*^ the two solutions of the every algebraic equation (24) or (28), using *Q*_*ψ*_^*I*^ and *Q*_*ψ*_^*II*^, and if we take into consideration that the SATBESPIG realizes inner pumping and exterior pumping, it is not possible to have a negative fluid flow rate from a physical point of view. Thus, the algebraic solution, which has physical meaning, is the positive solution [3, 5].

The numerical evaluation of *Q*_*ψ*_^*I*^ and *Q*_*ψ*_^*II*^ algebraic solutions, for *Q*_*ψ*_^*I*^ and *Q*_*ψ*_^*II*^ using medium (normal) values for the physical and geometrical dimensions [1, 2] of (24) and (28), leads to *Q*_*ψ*_^*I*^ < 0 and *Q*_*ψ*_^*II*^ > 0. Thus, the mathematical relation for the calculus of the fluid volumetric flow rate *Q*_*ψ*_ is

(30) $$\begin{array}{c} Q_{\psi }\equiv Q_{\psi }^{II}, \end{array}$$

where, in conformity to the devoted notations from the classical algebra, the solution *Q*_*ψ*_^*II*^ is

(31) $$\begin{array}{c} Q_{\psi }^{II}=\frac{-\ddot{b}-\sqrt{{\ddot{b}}^{2}-4\ddot{a}\ddot{c}}}{2\ddot{a}}, \end{array}$$

where $\ddot{a}$, $\ddot{b}$, and $\ddot{c}$ are the coefficients of the algebraic equations (24) or (28).

## 8. Numerical and Experimental Results 

The established mathematical relations allow the numerical calculation of the pressure and speed distributions for several SATBESPIG with inner or external pumping in permanent and laminar regimes. For these numerical calculations we used two different computer programs: one for the SATBESPIG with inner pumping and the other for the SATBESPIG with external pumping.

**Figure 6:**
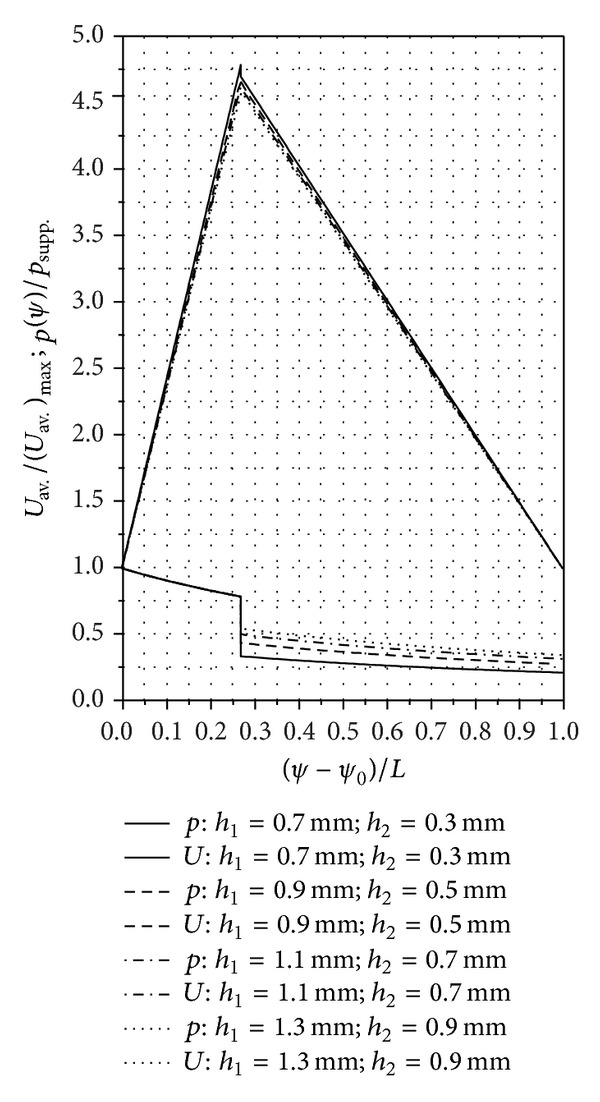
Pressure and speed distributions in the SATBESPIG with inner pumping. (See details in Figures 7 and 8.)

**Figure 7:**
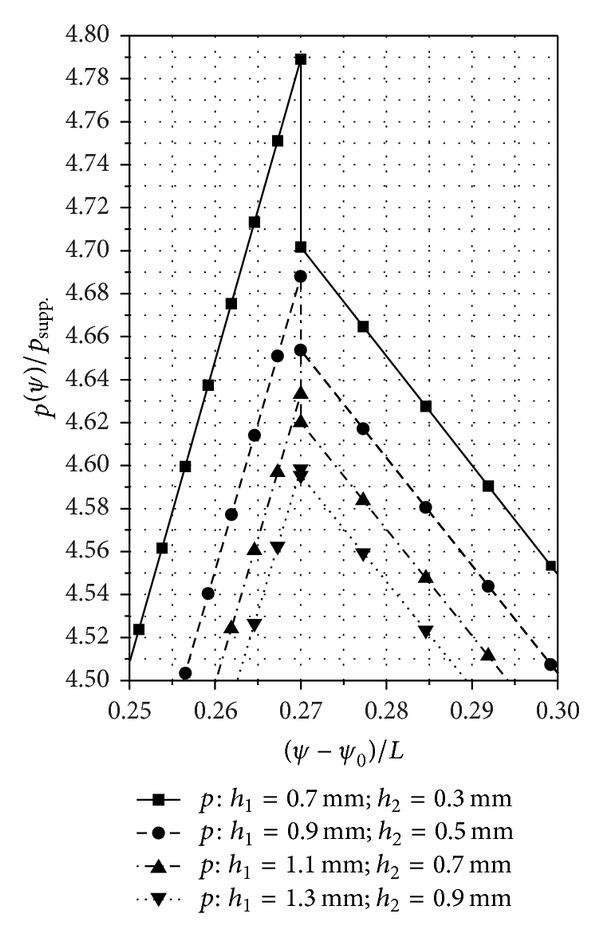
Detail of Figure 6 concerning the pressure jump between *h*_1_ and *h*_2_. (The marked points are those where we made the calculation.)

**Figure 8:**
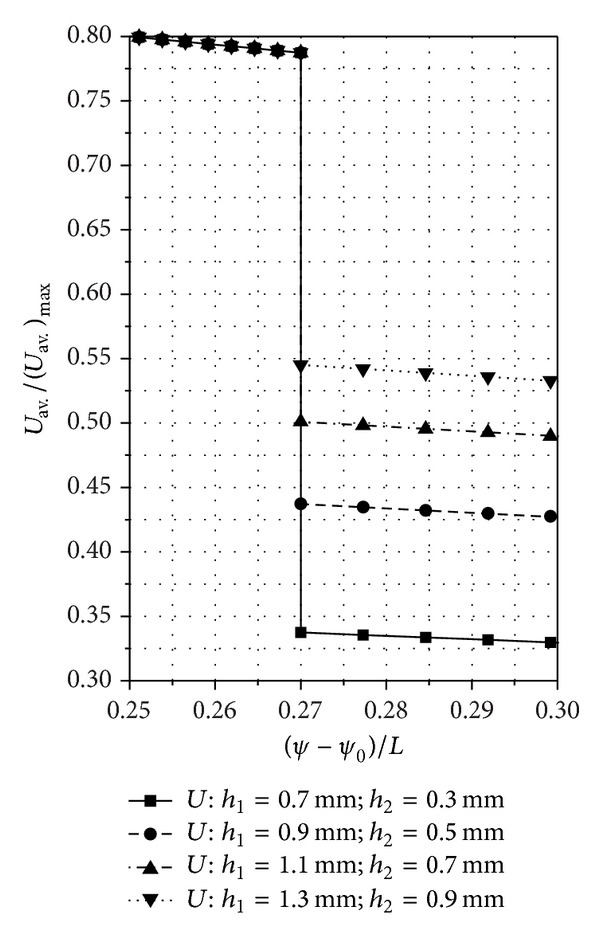
Detail of Figure 6 concerning the speed jump between *h*_1_ and *h*_2_. (The marked points are those where we made the calculation.)

In Figures 6 to 8, we present the calculated pressure and speed distributions for a SATBESPIG with inner pumping and *r*_*e*_ = 90 mm, *r*_*c*_ = 57.15 mm, *r*_*i*_ = 45 mm, *n*_*p*_ = 10, *α* = 0.615, *β*_0_ = 17°, **ρ** = 905 kg/m^3^, *η* = 10^−4 ^Pa·s, *p*_supp._ = 101325 N/m^2^, *n* = 900 rot/min, *α*_0_ = 6/5, *ε* = 2/15, *γ* = 1/5, *δ* = 1/10, *β* = 2/15, and the parameters *h*_1_ and *h*_2_ as written in the figures.

**Figure 9:**
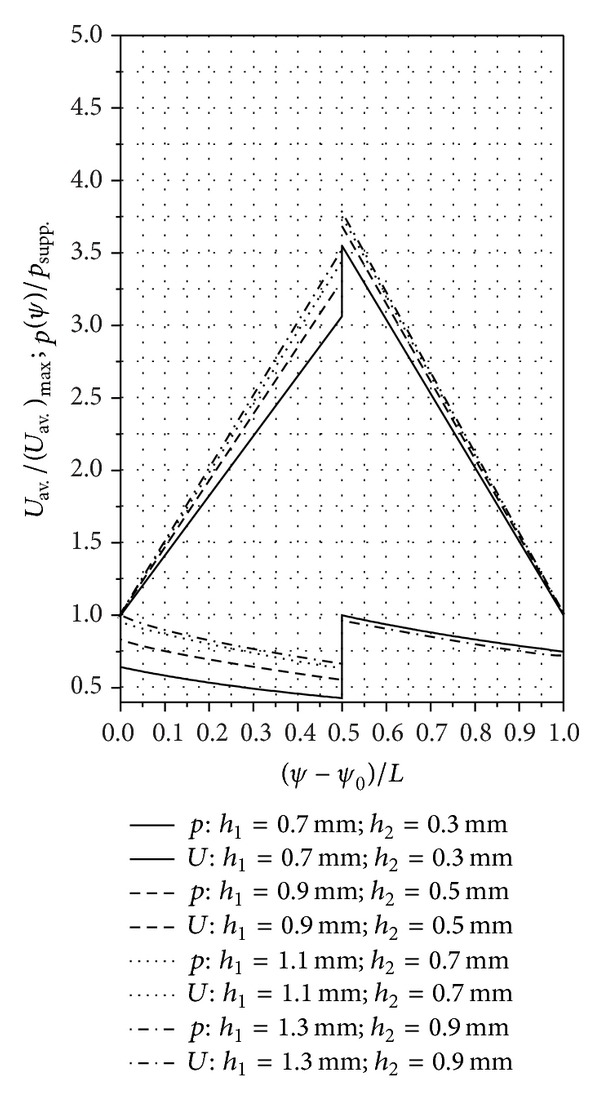
Pressure and speed distributions in the SATBESPIG with external pumping.

Figure 9 presents the calculated pressure and speed distributions for a SATBESPIG with external pumping with the same characteristics as the SATBESPIG with inner pumping above, with the exception that *r*_*c*_ = 77.85 mm.

**Figure 10:**
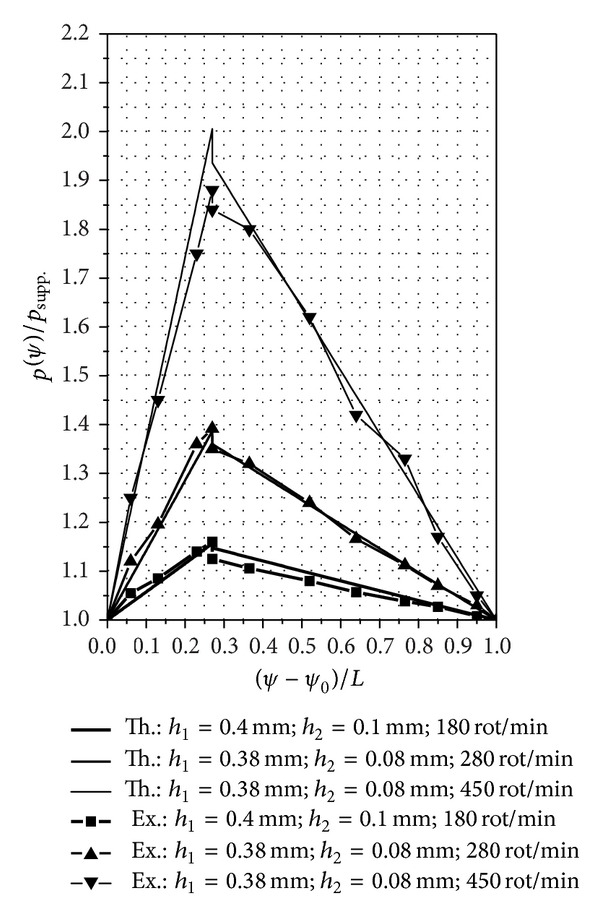
Pressure distributions in the SATBESPIG with inner pumping (comparison between the theoretical and experimental curves). The marked points are those where we performed the experimental measurements.

In Figure 10, we compared the theoretical results and the experimental measurements for the SATBESPIG with inner pumping and *h*_1_, *h*_2_, and *n* written in the figure.

In all our studies, the main constructive geometrical and functional parameters for the calculation of these bearings were *h*_1_, *h*_2_, *n*, *r*_*i*_, and *r*_*e*_ [3, 5].

## 9. Discussion and Conclusions

The analysis of the theoretical results allows some conclusions to be drawn concerning the laminar motion of incompressible fluids in some models of a SATBESPIG. We analyzed two variants of the SATBESPIG with inner pumping, which are called the First Variant and the Second Variant below. The difference between these bearings is the *r*_*c*_ dimension. The First Variant is the SATBESPIG from Figure 6 (*r*_*c*_ = 57.15 mm), and the Second Variant has *r*_*c*_ = 70.29 mm.

- For all the rotation per minute (r.p.m.) ranges *n* and for all the analyzed rapports *h*_1_/*h*_2_, the First Variant SATBESPIG with inner pumping had superior hydrodynamics performance compared to the Second Variant SATBESPIG. Thus, high pressures in bearings can be realized with an appropriate dimension of the bearing surface, especially the dimension of the grooved surface, meaning the parameters *r*_*i*_, *r*_*c*_, and *r*_*e*_.
- Generally speaking, increasing other hydraulics performance of the SATBESPIG for the same dimensions *r*_*i*_, *r*_*c*_, and *r*_*e*_ can be realized by increasing the number of the rotation per minute *n*. The modification of the rapport *h*_1_/*h*_2_ has a small influence.
- The nondimensional speeds increase (and decrease) from the bearing input to the bearing output, with a jump at the radial step *r*≅*r*_*c*_. The nondimensional pressures change, but not linearly with the channel length, from the input, where *p*_imput_≅*p*_supp._, up to the pressure *p*_*h*1_ and from the pressure *p*_*h*2_ to the output pressure, where *p*_output_≅*p*_supp._.
- The pressure jump Δ*p* = *p*_*h*1_ − *p*_*h*2_ theoretically tends to zero when the rapport *h*_1_/*h*_2_ → 1 and thus when the radial step vanishes or when *p*_*h*1_≅*p*_*h*2_. The case *p*_*h*1_≅*p*_*h*2_ appears only when we do not take into consideration the inertial effects.
- The pressure jump Δ*p* depends not only on the rapport *h*_1_/*h*_2_ but also on the value of the *n* and on the bearing geometry (*r*_*i*_, *r*_*c*_, and *r*_*e*_).
- For the same constructive geometrical and functional parameters (*r*_*i*_, *r*_*e*_, *h*_1_/*h*_2_, *n*,…), the SATBESPIG with exterior pumping gives lower pressures than the bearing with inner pumping.
- The comparative analysis between the theoretical and experimental results shows good correlation, especially at low *n* and at middle values for the rapport *h*_1_/*h*_2_ (*h*_1_/*h*_2_≅4). Some of the mathematical relations established above can be used to approach the theoretical functionality of the SATBESPIG having magnetic controllable fluids (magnetic fluids or magnetorheological fluids) as incompressible fluids, in the presence of a controllable magnetic field [18–20].
